# Pulmonary Artery Thromboembolism as a Complication of Essential Thrombocythemia

**DOI:** 10.7759/cureus.85839

**Published:** 2025-06-12

**Authors:** Ketevan Sulakvelidze, Nino Dolidze

**Affiliations:** 1 Department of Cardiology, Caucasus Medical Centre, Tbilisi, GEO

**Keywords:** essential thrombocythemia (et), hematologic disorder, hypercoagulability, myeloproliferative neoplasm, pulmonary embolism, pulmonary thromboembolism, thrombosis, venous thromboembolism

## Abstract

Essential thrombocythemia (ET) is a chronic myeloproliferative neoplasm characterized by elevated platelet counts and an increased risk of thrombotic or hemorrhagic complications. It typically affects older individuals, with a higher prevalence in women, and is frequently associated with Janus kinase 2 (JAK2) mutations. While many cases follow an indolent course, major thrombotic events such as pulmonary embolism may occur. We report the case of a 72-year-old female patient with a known history of ET who presented with an acute onset of dyspnea and pleuritic chest pain. Computed tomography pulmonary angiography confirmed pulmonary artery thromboembolism. Laboratory tests revealed significant thrombocytosis. The patient was treated with anticoagulation and cytoreductive therapy, resulting in clinical improvement. This case highlights the importance of early recognition of thrombotic complications in patients with ET, even when they are receiving ongoing treatment.

## Introduction

Essential thrombocythemia (ET) is a hematopoietic stem cell disorder characterized by excessive platelet production. It predominantly affects individuals over 50 years of age and is more commonly observed in women [1]. A significant proportion of cases are associated with Janus kinase 2 (JAK2) mutations, which belong to the tyrosine kinase family of enzymes and play a key role in signal transduction for erythropoietin, thrombopoietin, and granulocyte colony-stimulating factor. The presence of these mutations contributes to clonal thrombocytosis and increases the risk of thrombotic complications [2]. In addition to JAK2 V617F, mutations in calreticulin (CALR) and myeloproliferative leukemia virus oncogene (MPL) are also commonly involved, especially in JAK2-negative individuals. These mutations result in constitutive activation of the JAK-STAT signaling pathway, promoting megakaryocytic hyperplasia and uncontrolled platelet production. The resulting platelets often exhibit qualitative abnormalities, which contribute to both thrombotic and hemorrhagic tendencies.

Thrombosis in ET can affect both the arterial and venous systems, but arterial events are more frequent. Arterial thrombosis, often affecting cerebral and coronary vessels, is primarily driven by platelet activation and endothelial interaction. Overexpression of P-selectin and CD40 ligand (CD40L) on activated platelets enhances platelet-leukocyte aggregates and promotes endothelial activation, fostering a pro-inflammatory and pro-thrombotic state. Endothelial cells, when activated by these mediators, increase expression of adhesion molecules and tissue factor, further amplifying the coagulation cascade. In contrast, venous thrombosis, such as deep vein thrombosis (DVT) or splanchnic vein thrombosis, is more associated with blood stasis and increased plasma coagulability. While both forms of thrombosis share overlapping mechanisms, the prominence of platelet-driven inflammation distinguishes arterial events.

Furthermore, patients with ET often exhibit elevated levels of thromboxane A2, contributing to vasoconstriction and platelet aggregation, as well as reduced levels of nitric oxide and prostacyclin from endothelial cells, which normally act as inhibitors of platelet activation. Although ET is often indolent in nature, it can be complicated by thrombosis, hemorrhage, and microvascular disturbances such as headaches, lightheadedness, or acral paresthesia. Platelet dysfunction, despite elevated counts, contributes to bleeding risk due to defective aggregation responses and altered granule release. In rare cases, disease progression to myelofibrosis or acute myeloid leukemia may occur [3]. This case underscores the thrombotic potential of ET and highlights the need for a high index of suspicion when evaluating patients with respiratory symptoms, even if they are undergoing treatment.

## Case presentation

A 72-year-old Caucasian woman presented to the emergency department in Tbilisi, Georgia, with dizziness, vomiting, chest discomfort, fatigue, muscle weakness, exertional dyspnea, and palpitations. Her medical history included hypertension and ET. She had been receiving hydroxyurea as first-line cytoreductive therapy; however, over the past month, she experienced worsening symptoms, including increased fatigue, dyspnea, and rising platelet counts, despite treatment. In response to suboptimal disease control and possible intolerance to hydroxyurea, her hematologist initiated ruxolitinib (15 mg twice daily). She was also maintained on aspirin (cardiomagnyl 75 mg daily) for antiplatelet prophylaxis.

On examination, the patient was afebrile (36.7°C), with blood pressure 120/70 mmHg, heart rate 95 beats per minute, and respiratory rate 28 breaths per minute, and oxygen saturation was 87% on supplemental oxygen administered via a face mask at a moderate flow rate of 6-8 liters per minute. Cardiovascular exam was unremarkable. Respiratory assessment revealed decreased breath sounds bilaterally. Abdominal and genitourinary examinations were non-contributory. Neurologically, she was alert and oriented.

**Figure 1:**
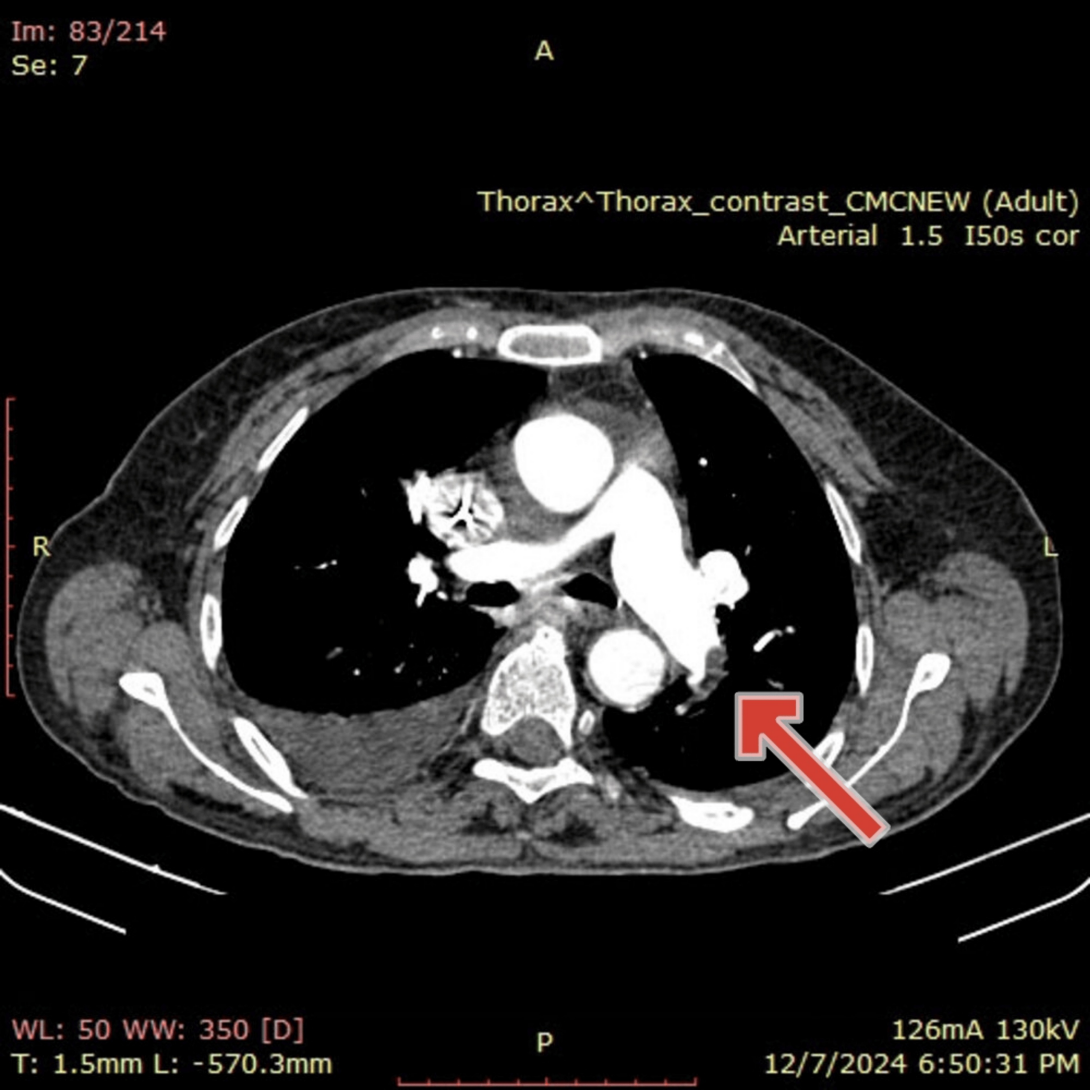
CT pulmonary angiography demonstrating pulmonary embolism CT pulmonary angiography revealed a central filling defect within the right pulmonary artery, consistent with an acute pulmonary embolism. The embolus appears as a sharply demarcated hypoattenuating area within the contrast-enhanced vessel, confirming the presence of an obstructive thrombus. The red arrow highlights this filling defect, which is located in a segmental or lobar branch of the right pulmonary artery, based on anatomical orientation and distribution.

Computed tomography pulmonary angiography (CTPA) revealed thrombi in the lower pulmonary lobes and the left upper lobe, confirming pulmonary artery thromboembolism, as shown in Figure 1. Her Pulmonary Embolism Severity Index (PESI) score was 117 (Class IV), indicating a 30-day mortality risk of 4.0-11.4% [4]. This score guided the decision for inpatient management and close monitoring, and while thrombolysis was not required due to hemodynamic stability, it reinforced the urgency of initiating anticoagulation and involving a multidisciplinary team. A chest ultrasound was performed to assess for pleural effusion and revealed bilateral fluid accumulation, supporting the clinical suspicion of heart failure as a contributing factor to the patient’s respiratory symptoms. Chest ultrasound showed bilateral pleural effusion. A lower limb Doppler ultrasound was performed to identify the source of DVT and revealed thrombosis in the left antero-posteromedial veins as well as the medial and lateral groups of the sural veins. Abdominal CT revealed mild ascites and signs of anasarca, consistent with heart failure. A repeat chest X-ray showed improvement in a right lower lobe fibrotic infiltrate.

Laboratory tests, as shown in Table 1, revealed markedly elevated high-sensitivity troponin I at 1263.10 ng/L (normal <34 ng/L), indicating myocardial strain. Platelet count was significantly elevated at 1539 × 10⁹/L (normal range: 150-400 × 10⁹/L), consistent with thrombocytosis. White blood cell count was 49.8 × 10⁹/L (normal range: 4.0-10.0 × 10⁹/L), indicating leukocytosis. Hemoglobin was elevated at 156 g/L (normal range: 121-151 g/L for women), and prothrombin time (PT) was prolonged at 16.9 seconds (normal range: 10.6-13.7 seconds). The fibrinogen level was elevated at 4.95 g/L (normal range: 2.0-4.0 g/L), and C-reactive protein (CRP) was markedly raised at 87.53 mg/L (normal <5 mg/L) [5].

**Table 1:** Laboratory findings PT: Prothrombin time; aPTT: activated partial thromboplastin time

Test	Admission Result	Discharge Result	Normal Range
High-Sensitivity Troponin I	1263.10 ng/L	38 ng/L	< 34 ng/L
RBC	8.1 × 10¹²/L	6.47 × 10¹²/L	4.2 – 5.4 ×10¹²/L
HGB	156.0 g/L	130.0 g/L	121 – 151 g/L
HTC	52.60%	41.70%	36 – 47%
PLT	1539 × 10⁹/L	792 × 10⁹/L	150 – 400 ×10⁹/L
C-reactive protein	87.53 mg/L	17.540 mg/L	< 5 mg/L
WBC	49.8 × 10⁹/L	20.4 × 10⁹/L	4.0 – 10.0 ×10⁹/L
PT	16.9 sec	14.2 sec	10.6 – 13.7 sec
aPTT	37.1 sec	32.3 sec	22.1 – 28.1 sec
Fibrinogen	4.95 g/L	3.8 g/L	2.0 – 4.0 g/L

The patient received supplemental oxygen, intravenous heparin (25,000 U/5 mL) with activated partial thromboplastin time (aPTT) monitoring, hydroxyurea 500 mg twice daily, captopril 50 mg for blood pressure management, and empirical piperacillin/tazobactam 4.5 g (4 g piperacillin + 0.5 g tazobactam). Empirical antibiotics were initiated due to markedly elevated inflammatory markers, respiratory symptoms, and the presence of pleural effusion, raising concern for possible secondary pulmonary infection in the context of an immunocompromised state. She developed atrial fibrillation during hospitalization, which was managed with intravenous amiodarone and successfully treated with pharmacologic cardioversion. By discharge, her vitals had stabilized and lab results improved (PLT 792 × 10⁹/L; WBC 20.4 × 10⁹/L).

She was discharged in stable condition with instructions to continue anticoagulation and cytoreductive therapy and follow up with hematology and cardiology.

## Discussion

ET is a chronic myeloproliferative disorder associated with increased platelet production due to mutations in JAK2, CALR, or MPL genes. While arterial thrombosis is more common in ET, occurring in approximately 20-25% of patients, venous thromboembolism (VTE) also occurs in about 7-12% of cases and must be considered in symptomatic patients. Arterial events, such as strokes, transient ischemic attacks, and myocardial infarctions, tend to predominate due to platelet-driven mechanisms, whereas venous thrombosis, including DVT and pulmonary embolism, though less frequent, carries significant morbidity and is more often observed in JAK2-positive individuals. The JAK2 V617F mutation, seen in 50-60% of ET cases, is associated with increased thrombotic risk [1,2]. Extreme thrombocytosis can paradoxically increase bleeding risk due to acquired von Willebrand disease [3].

Risk stratification for thrombosis in ET includes age, prior thrombotic events, and JAK2 mutation status [4]. Our patient met high-risk criteria due to her age and JAK2-positive status. Though VTE is less commonly associated with ET, some cases may involve masked polycythemia vera, particularly in elderly women, making differential diagnosis essential [5].

In this case, pulmonary embolism was confirmed on CTPA with a PESI score placing the patient in Class IV. The management strategy included anticoagulation and cytoreductive therapy, both of which resulted in clinical improvement. In patients with ET who develop VTE, anticoagulation is typically recommended for at least 3 to 6 months, with longer or indefinite duration considered in high-risk cases-such as those with persistent thrombophilia, ongoing myeloproliferative activity, or recurrent thrombotic events. Choice of anticoagulant may vary; while vitamin K antagonists like warfarin (Coumadin) have historically been used, direct oral anticoagulants such as rivaroxaban or apixaban are increasingly preferred due to their predictable pharmacokinetics, lack of need for routine INR monitoring, and lower risk of intracranial hemorrhage. However, evidence in ET-specific populations is still evolving. Heparin-based anticoagulation, particularly low-molecular-weight heparin, is often used in the acute phase or when rapid anticoagulant effect is needed, with bridging to oral agents depending on clinical status and bleeding risk. In our patient, initial treatment involved intravenous heparin, followed by planned transition to long-term oral anticoagulation. The elevated PESI score of 117, which incorporates factors such as advanced age, tachypnea, low oxygen saturation, and comorbid conditions, indicated a 30-day mortality risk of 4.0-11.4%. This prompted inpatient admission to a monitored setting and early multidisciplinary involvement. Cardiology was consulted due to elevated troponin levels and new-onset atrial fibrillation; hematology managed cytoreductive treatment adjustments; and internal medicine oversaw overall coordination of care. Hydroxyurea remains the first-line therapy in high-risk ET patients [6,7]. However, when patients show signs of clinical decline, rising platelet counts, or intolerance to hydroxyurea, treatment may be escalated. In this case, the patient had experienced progressive fatigue, dyspnea, and thrombocytosis over the preceding month, leading her hematologist to initiate ruxolitinib, a JAK1/2 inhibitor, as second-line therapy. Overall, this case illustrates the need for both diagnostic vigilance and collaborative treatment strategies in addressing the less common but serious venous manifestations of ET.

## Conclusions

Pulmonary embolism is an uncommon but serious complication of ET. Though ET is predominantly linked to arterial events, clinicians should maintain suspicion for VTE in symptomatic patients. Prompt diagnosis and treatment with anticoagulation and cytoreduction are essential for reducing morbidity and improving outcomes. Long-term management should include regular hematologic follow-up to monitor platelet counts, assessment of thrombotic and hemorrhagic risks, and adjustment of cytoreductive therapy as needed. In addition, cardiovascular risk factors should be carefully managed, and patients should be educated on symptom recognition and medication adherence. Preventive strategies, including antiplatelet therapy and lifestyle modifications, play a critical role in minimizing recurrent thrombotic events and ensuring better long-term prognosis.
